# Correction

**DOI:** 10.1093/ofid/ofae247

**Published:** 2024-05-10

In the originally published version of the below-listed abstracts, the name of Bradford D Gessner was incorrectly listed as Bradford J Gessner.

This has been corrected.

**https://doi.org/10.1093/ofid/ofac492.638**

**https://doi.org/10.1093/ofid/ofac492.1826**

**https://doi.org/10.1093/ofid/ofac492.1289**

**https://doi.org/10.1093/ofid/ofac492.449**

**https://doi.org/10.1093/ofid/ofad500.2218**

**https://doi.org/10.1093/ofid/ofad500.933**

**https://doi.org/10.1093/ofid/ofad500.2242**

**https://doi.org/10.1093/ofid/ofad500.050**

**https://doi.org/10.1093/ofid/ofad500.096**

**https://doi.org/10.1093/ofid/ofad500.990**

**https://doi.org/10.1093/ofid/ofad500.937**

**https://doi.org/10.1093/ofid/ofad500.332**

**https://doi.org/10.1093/ofid/ofad500.2234**

